# Case of Injury of the Hip Confounded with Dislocation, and Fracture of the Neck of the Thigh-Bone

**Published:** 1815-06

**Authors:** John C. Yeatman

**Affiliations:** Member of the Royal College of Surgeons, &c. Frome


*For the Medical and Physical Journal.*

### *Case of Injury of the Hip confounded with Dislocation, and Fracture of the Neck of the Thigh-Bone;* by Mr. JOHN C. YEATMAN, of Frome.

IT is by no means rare to find surgeons baffled in their opinions concerning the nature of certain injuries to which the human body is liable; and, as this leads them to differ on the plan of treatment, it is of most importance to record even a single case in which there is a difficulty of pronouncing a judgment.

A private of a regiment of foot, æt. 38, robust, fell from the top of an embankment upon the left buttock. He was carried to the Regimental Hospital immediately after receiving the injury, when I saw him.

The whole of the left buttock was considerably enlarged, particularly behind the usual situation of the great trochanter. This enlargement or swelling was attended with slight discolouration of the skin, and hard to the touch. The limb was shorter by about three-quarters of an inch than its fellow, was drawn forward and upward in an oblique direction towards the opposite thigh, with the foot turned inward. The patient had no power to move the limb, nor did he complain complain of pain when it was moved by me. The great trochanter was not to be distinguished, in consequence of the swelling.

After some consideration, I regarded the case as a muscular injury, in which several or all of the extensors and abductors, and more particularly the rotators outwards, had partially or wholly lost their tone by the blow. In that event, the thigh would, doubtless, be drawn into the position above described, and be retained therein by the flexors, abductors, and rotators, inwards—the antagonists of the former sets of muscles. This opinion admitted, the man would be unable to move his limb, and no particular pain would ensue on its being moved by another. The swelling of the buttock might have arisen from extravasation of blood between the layers and fibres of the glutei muscles, and all the circumstances of the case in this way explained.

The surgeon of the battalion, however, thought it a dislocation of the femur, and took the usual means to effect its reduction, which produced no other consequence than an increase of the subsequent inflammation. A third medical friend was disposed to believe it a fracture of the neck of the thigh-bone.

Now, supposing the head of the femur had been thrown upward and outward upon the dorsum of the ilium, where the swelling was situated, the limb would have been from two to three inches shorter than before, and could not have been moved without difficulty. If it had been dislocated upward and backward, with the trochanter major forward, and the head of the bone backward, the same would have occurred, with this addition, that the patient would have experienced great pain on my moving the thigh-bone outward, and the fibres of the triceps would have been found upon the stretch; in both instances a hollow would have been discoverable in the situation of the acetabulum; indeed, great pain, immobility, and a far more considerable shortening of the limb, than took place in this case, were sufficient to distinguish it from any kind of dislocation to which it bore a similitude. And, on the other hand, had it been a fracture of the neck of the femur, the foot could have been turned outward, and crepitus, mobility, and extreme pain, would have been the attendant circumstances.

The treatment and result of the case, however, left no doubt on our minds as to its real nature; and, on this head, it will be enough to say, that bleeding, cold applications, saline purgatives, low diet, &c. were resorted to during the inflammation which ensued, and that blisters and other local stimuli stimuli were subsequently applied to the injured muscles. The swelling subsided, the limb gradually returned to its natural position, and in the course of a few months the patient was able to walk. JOHN C. YEATMAN,

*Member of the Royal College of Surgeons, &c.*

*Frome; May 8th,* 1815.

*Memorandum.*—Two cases of recovery from an immoderate dose of laudanum, analogous to that which I narrated in No. 184 of the Med. and Phys. Journal, are recorded in the Journals for March and the present month. Mr. M. Rowe, the author of one of them, adds, "From an attentive observation of this case, and of the symptoms which were manifested throughout, I am induced to believe, owing to the sedative powers of opium, that, had I had recourse to bleeding, (as recommended by Cullen and others,) it would not only have considerably retarded, but might eventually have endangered, the recovery of my patient." It gives me pleasure to notice that these cases and this opinion tend to strengthen the observations which, with other matter, are contained in my paper for August.—Vide No. 186, Med. and Phys. Journ.

I have been induced to remind your readers in this way of the importance of communicating the result of their practice in these cases, in order that we may in future have recourse to a safer and more settled plan of treatment than we have hitherto had in respect to them. J. C. Y.

---

